# The evolving landscape: magnetic resonance imaging in active surveillance for prostate cancer management

**DOI:** 10.3389/fruro.2024.1329274

**Published:** 2024-04-12

**Authors:** Irene De la Parra, Juan Gómez Rivas, Beatriz Gutiérrez, María Jesús Marugán, Dmitry Enikeev, Bhaskar K. Somani, Jerónimo Barrera, Jesús Moreno-Sierra

**Affiliations:** ^1^ Department of Urology, Health Research Institute, Clínico San Carlos Hospital, Complutense University, Madrid, Spain; ^2^ Institute for Urology and Reproductive Health, Sechenov University, Moscow, Russia; ^3^ Department of Urology, University Hospital Southampton, Southampton, United Kingdom; ^4^ Department of Radiology, Clínico San Carlos Hospital, Complutense University, Madrid, Spain

**Keywords:** prostate cancer, active surveillance, magnetic resonance imaging, overdiagnosis, overtreatment, PRECISE

## Abstract

Since overdiagnosis and overtreatment pose significant risks in managing prostate cancer (PCa), active surveillance (AS) is the most common treatment in low-risk patients. However, there is no general agreement yet on the inclusion criteria and the required follow-up. Multiparametric magnetic resonance imaging (mpMRI) of the prostate was born as a useful device in these patients both in diagnosis and follow-up, and it is widely used in daily clinical practice. We reflect on the most current evidence described in the literature on the topic, its results, and our experience.

## 1 Introduction

Overdiagnosis and overtreatment of indolent prostate cancer (PCa) in aging men pose significant risks in the management of PCa (1) due to the prevalence of the disease, the usual accessibility of the prostate-specific antigen (PSA) test, and the long-term effects of radical treatments. In the early 2000s, active surveillance (AS) became a strategy in the management of PCa patients, and nowadays, AS is extensively used and adopted (2).

The inclusion criteria for AS have varied among groups and their diversity demands more comparisons of studies. Multiparametric magnetic resonance imaging (mpMRI) has a developing role in early staging to enhance the candidate’s selection for AS by proposing a visual staging of the gland and determining areas to be selected for image-guided biopsies. The objective of our paper is to assess the current role of mpMRI in the different AS scenarios.

## 2 Materials and methods

We developed a literature search in PubMed and Medline with the following words: prostate cancer, active surveillance, PRECISE score, and multiparametric magnetic resonance imaging. We found more than 60 articles, from which we selected the 20 with the most scientific interest after consensus among the authors. Moreover, we also describe the application of mpMRI in our centre within the recent PRECISE score developed to follow up on patients in AS protocols.

## 3 Results

mpMRI is widely used worldwide in different scenarios within the AS protocol, as reflected in numerous articles published in high-impact journals in the last 5 years.

### 3.1 Evidence synthesis

There is a life expectancy of 10–15 years for any radical treatment in a localised disease. What AS aims is to evade unnecessary invasive treatments and treatment-related side effects in men with clinically localised PCa who do not need rapid therapy and to appropriately determine the need for such therapy for those who need it. Patients are kept under systematic follow-up, and therapy is guided by predetermined cutoff points denoting a potentially life-threatening disease. During the follow-up, more than 33% of patients who were “re-categorised,” most of whom underwent therapy for different reasons such as disease upgrading, a rise in the extent of the disease, disease progression or patient’s decision (1).

Two different approaches guide the conservative management of localised PCa. On the one hand, AS is used for men whose age and health condition make them candidates for radical treatment, to be monitored over time, and reclassified if they have high-risk for disease progression according to clinical and disease features. On the other hand, watchful waiting (WW) involves minimal follow-up with intentions of palliative therapy for those with advanced disease and for whom extreme treatment is not an option because of age and comorbidity (**Table 1**) (1).

**Table 1:** Differences between AS and WW according to the National Cancer Comprehensive Network (NCCN) and the European Association of Urology (EAU) Guidelines (3, 4).

	Treatment intent	Markers	Inclusion criteria	Follow-up	Advantages	Disadvantages
AS	Curative	Digital rectal examination (DRE), PSA, re-biopsy, mpMRI	Mainly low-risk patients with >10 years of life expectancy	Predefined schedule	Side effects of definitive treatment that might not be necessary will be avoided	- Probability of missed curative treatment, although very low - Systematic follow-up, mpMRI, and prostate biopsies might be required
WW	Palliative	Not stabilised	Applicable to patients in all stages with <10 years of life expectancy	Patient-specific	Side effects of unneeded definitive treatment and androgen deprivation therapy (ADT) will be avoided	- Possibility of urinary retention or pathologic fracture without prior symptoms or concerning PSA levels

The first studies on expectant management were led by the Prostate Cancer Research International Active Surveillance (PRIAS) study, which started in 2006 from the European Randomised Study of Screening for Prostate Cancer (ERSPC). It is a web-based prospective, observational study, which has spread internationally, and it intends to present a real context with contributions from academic, nonacademic, and private practices around the world, enlarging the universalisation of the results (5, 6). Eventually, it became the broadest prospective AS study worldwide with >150 participating centres in 18 countries (7).

The PRIAS protocol has varied over the years, incorporating possible targeted biopsies (TBs) in 2013. Due to these changes, the 2006 inclusion criteria (Gleason grade (GG) ≤1, clinical stage ≤T2c, PSA ≤10 ng/mL, ≤2 cores positive for PCa, PSA density ≤0.2 ng/mL/cm (3), and fitness for therapy) have been modified by allowing patients with >2 cores positive for PCa in the PRIAS study where MRI is used before diagnosis or during follow-up. Classically, regular PSA and digital rectal examination (DRE) are carried out, and a repeat biopsy is advised 1, 4, 7, and 10 years after inclusion. Since 2013, performing an MRI with TB 3 months after inclusion, combined with systematic biopsies (SBs) after 1, 4, 7, and 10 years has been recommended (6).

Regarding AS discontinuation, in 2016, Bokhorst et al. (6) found that only 50% of the men enrolled in the study were still on AS after 5 years of follow-up and only 25% after 10 years of follow-up in the PRIAS study. In this respect, Luiting HB et al. (8) recently analysed how MRI has contributed to the number of patients discontinuing AS in the PRIAS study. They were differentiated into three groups based on the use or nonuse of MRI: (1) nonuse of MRI (Group A), (2) use of MRI during AS but not before diagnosis (Group B), and finally, (3) the use of MRI prior to diagnosis and during AS (Group C). They showed that using MRI with feasible TB before the inclusion in AS lowers the chance of discontinuing AS after 2 years, because of a stricter selection at the beginning of AS. Moreover, the use of MRI in patients already in AS increases the chance of discontinuing AS because of increased GG re-categorisation. This leads to the acknowledgment that this higher re-categorisation rate might increase overtreatment, and so new definitions for clinically significant PCa after MRI may be needed.

Our centre has collaborated with the PRIAS study since 2021. At the moment, we have identified 65 patients in AS in our centre. The average age at diagnosis was 66 years, a PSA of 5.6, and a Charlson index of 3 [IQR 2–3]. Of the 65 patients, 98.5% had an ISUP grade 1 PCa based on the International Society of Urological Pathology (ISUP) grading, with a mean of 13 cores obtained in a prostate biopsy, and 79% having one or two positive cores. Of these, 56 (98%) underwent a first re-biopsy, with a mean PSA of 5, all with previous mpMRI, and for 66.7% of them, results of the mpMRI showed a lesion, with a score of 3 or 4 in the Prostate Imaging-Reporting and Data System (PI-RADS). More than half of the patients (60%) underwent a transperineal fusion biopsy with a mean of 5 cores taken from the target and 16 cores in the systematic biopsy, with 85% being negative for malignancy. The mean time to re-biopsy was 507 days. During this time, 13 (20%) patients have abandoned the protocol, 76% of them due to clinical progression (9).

Nowadays, the most recent National Comprehensive Cancer Network (NCCN) and the European Association of Urology (EAU) guidelines advise AS as a feasible choice for the management of low-risk PCa (3, 4, 10, 11). Regarding intermediate-risk PCa, the use of AS is debated. To evaluate prognosis better, cancer risk has been stratified into “favourable” and “unfavourable” subgroups (12). Since 2021, the EAU guidelines have recommended offering AS to carefully chosen patients with an ISUP grade 2 disease, acknowledging the heightened potential risk of metastatic progression. NCCN guidelines allow AS in favourable subgroups of intermediate-risk patients. Enikeev et al. (13) summarised the outcomes of AS in these patients and concluded that AS could be offered; nevertheless, they should be notified about the need for regular supervision and the choice of discontinuation. Available data show that 5-year survival rates in intermediate-risk patients are similar to those in low-risk patients, whereas 10-year survival rates are poorer.

We note three prominent randomised trials that have compared the outcomes of expectant management to radical prostatectomy. The three studies are the Scandinavian Prostate Cancer Group Study Number 4 (SPCG-4) (14), the Prostate Testing for Cancer Treatment (ProtecT) (15), and the Prostate Cancer Intervention versus Observation Trial (PIVOT) (16, 17).

The SPCG-4, carried out in the era before PSA, used the incidence of both metastasis and palliative treatment as literature about the extent of the disease, corroborating a substantial decline in mortality rate after radical prostatectomy and supporting AS as an alternative in selected groups (14). Regarding the ProtecT trial, they found that prostate cancer–specific mortality was low at a median follow-up of 15 years, regardless of the therapy designated (active monitoring, prostatectomy, or radiotherapy) (15). With regard to the active-monitoring group, there were 133 men (24.4%) who survived with no prostate cancer therapy at the end of follow-up. Active monitoring has some differences with contemporary AS protocols, based on PSA measurement alone, without the use of mpMRI, and without any protocol for repeating biopsies during regular follow-up (15). Finally, in the PIVOT trial (17), they did not associate radical prostatectomy with considerably lower all-cause or prostate-cancer mortality than observation through 19 years of follow-up. Moreover, surgery brought about larger long-term urinary incontinence and erectile dysfunction than observation and was linked to a considerably lower danger of disease progression and additional therapies. This trial did not include the mpMRI either. Three years later, Wilt et al. described all-cause mortality through 22 years, and their results confirmed that observation and PSA-based monitoring end in similar long term survival with less complications with surgery for men with PSA-detected low-risk PCa and many with intermediate or high-risk disease. Surgery was associated with a relative decrease of 8% of all-cause mortality in comparison with observation in men with clinically localised prostate cancer and a mean survival increase of 1 year (18). Klotz et al. (19) published their long-term results after 15 years of follow-up of an active surveillance cohort of 903 patients. In this study, only 2.8% of patients developed metastatic disease, and 1.5% died of prostate cancer, proving that AS for low-risk prostate cancer is feasible and seems safe in the 15-year time frame.

AS inclusion criteria have varied among groups and their diversity certainly demands more comparisons of studies (**Table 2**). Ordinary selection criteria for AS contain GG group 1, less than one-third to one-fourth positive cores with <50% of involvement, cT1c-T2a, PSA <10 ng/mL, and PSA density (PSAD) <0.15 ng/mL/cc (24, 25). The outcomes for patients with intermediate-risk (Gleason grade group 2) are opposing; some studies presume that determined patients might be suitable candidates that can be managed safely (26, 27), whereas others advocate that the risk of failure is considerably higher in comparison with low-risk patients (28). The concern of undergrading the tumour at the time of diagnosis biopsy has led to the improvement of AS protocols with rigorous criteria for inclusion and monitoring. mpMRI has a prominent role in early staging to refine the selection or exclusion of candidates for AS by suggesting a visual staging of the gland and determining locations to be selected for image-guided biopsies (29).

**Table 2:** Available outcomes of the five most important AS cohorts (20).

	JHU (21)	PASS (22)	PRIAS (6)	UCSF (23)	UT (19)
Inclusion criteria	PSA ≤ 10 ng/mL, PSAD ≤ 0.15 ng/mL, stage≤ T2a, Grade≤ 3 + 3, % positive cores ≤ 2, single-core positivity ≤ 50%	PSA ≤ 20 ng/mL, PSAD ≤ 0.15 ng/mL, stage≤T2c, Grade ≤ 3 + 4, % positive cores ≤ 1/3	PSA ≤ 10 ng/mL, PSAD ≤ 2 ng/mL, stage ≤ T2a, Grade ≤ 3 + 3, % positive cores ≤2	PSA ≤ 10 ng/mL, stage ≤T2a, Grade ≤ 3 + 3, % positive cores ≤1/3, single-core positivity ≤50%	PSA ≤ 10 ng/mL or PSA 10–20 ng/mL if LE <10 years, Grade ≤3 + 3 or Grade<3 + 4 if LE < 10 years
Role of MRI	Utilised but not incorporated into surveillance protocol	Not defined	At diagnosis and during follow-up	Not defined	Not defined
Median follow-up	5.0 years	2.3 years	1.6 years	5.0 years	6.4 years

JHU, Johns Hopkins University; PASS, Prostate Cancer Active Surveillance Study; PRIAS, Prostate Cancer Research International: Active Surveillance; UCSF, University of California San Francisco; UT, University of Toronto; and LE, life expectancy.

#### 3.1.1 The role of MRI during AS

Multiparametric MRI alludes to the application of diverse anatomical and functional imaging parameters, each of which depicts a particular feature of the prostate gland (30):

-   T2-weighted imaging (T2): It is the most helpful procedure to analyse the anatomy of the gland. The peripheral area looks bright because of the large presence of glandular tissue whereas the transitional area presents a heterogenous aspect with numerous stromal nodules made of muscle fiber bundles. Tumours on T2 display low signal intensity (31).
-   Diffusion-weighted magnetic resonance imaging (DWI): It evaluates the movement of water molecules within tissues. In PCa, long b-value sequences present a higher signal intensity (bright areas) while there is a lower signal (dark areas) on a reconstructed apparent diffusion coefficient (ADC) map (32). It has been seen that low ADC values correlate with higher Gleason-grade tumours on AS (33).
-   Dynamic contrast-enhanced (DCE) sequences refer to the intravenous dispensation of a particular contrast, mostly gadolinium. PCa often displays early washin and washout, because of its disordered vascularity (34). When there is focal, earlier or contemporaneous enhancement bordering on normal prostatic tissues, DCE is considered positive and frequently correlates with a suspicious discovery on T2 and/or DWI.

**Figure 1:**
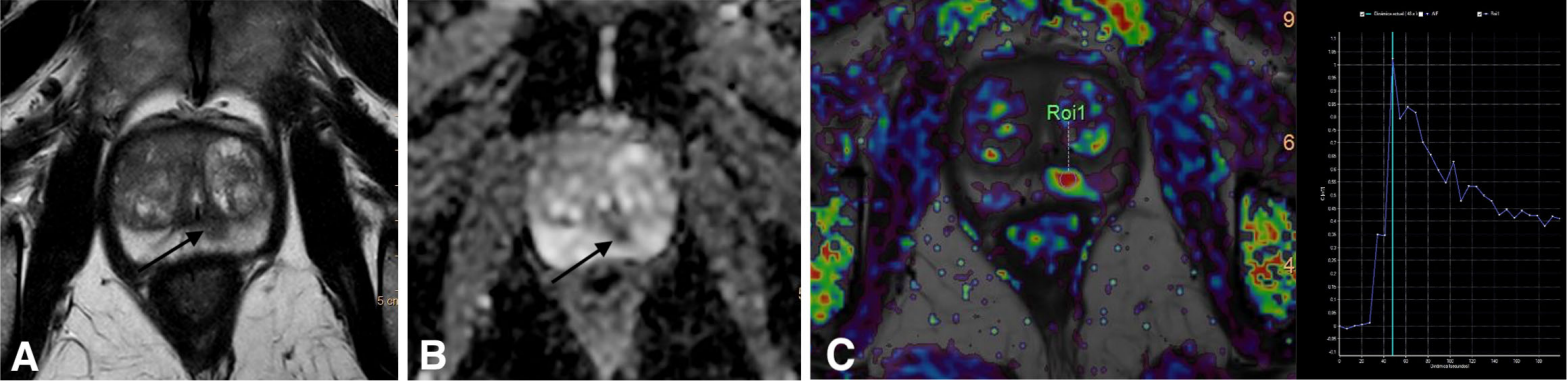
A PI-RADS 4 lesion on the posterior peripheric area of the prostate. **(A)** Axial T2-weighted sequence: T2 hypointense lesion on the posterior peripheric area of the prostate. **(B)** Axial diffusion-weighted sequence (ADC maps): focal restricted diffusion lesion. **(C)** Dynamic contrast-enhanced sequence (functional T1 perfusion maps) with early enhancement and contrast washout of the lesion.

The Prostate Imaging-Reporting and Data System (PI-RADS) is the most broadly recognised reporting system which was updated with version 2.1 in 2019 and it categorises lesions on a scale of 1–5. A score of 1 indicates that the presence of clinically significant cancer is very improbable and a score of 5 indicates that the presence of clinically significant cancer is very probable (35–37). **Figure 1** shows a PIRADS 4 lesion in the different MRI sequences. MRI images provide supplementary data on loco-regional staging, and whole gland imaging is particularly helpful in recognising anterior diseases, which cannot be determined by a rectal examination and widely missed by standard transrectal ultrasound (TRUS)-guided biopsy (38).

Currently, patients with low-risk PCa who initially chose AS are progressively assessed using MRI and targeted biopsy (39). The supplementary use of a first pre-biopsy MRI and successive target biopsy in these patients may help in the rejection of higher-risk ones with an ISUP grade ≥2 PCa, regardless of the schedule of the MRI (at baseline, confirmatory, or surveillance biopsy) (40, 41).

##### 3.1.1.1 MRI at the time of initial diagnosis

A recurrent concern is the fear of misclassifying PCa risk groups at the first systematised biopsy, resulting in overlooking high-risk cancer. Misclassification rates could be anywhere from 20% to 30% (42). Early re-biopsy results lead to a growth of 27% of cases with a Gleason score of ≥7 and negative findings in a similar number of patients (2). For this reason, undergoing a confirmatory biopsy within 1 year, and then systematic surveillance biopsies are usually recommended (43).

It is widely acknowledged that the negative predictive value of mpMRI for the identification of clinically significant cancer is very high (44) and it is reliably reported to be more than 90% (45). However, some tumours do not show on mpMRI due to a sporadic upgrading pattern and a low malignant epithelium-stroma proportion (39). Since mpMRI is known to have less sensitivity for locating low-volume Gleason 3 + 3 diseases, negative MRI findings could be a good predictor of the relevance of AS enrolment (46).

Recently, Robinson et al. (47) demonstrated, in their study of a cohort of 23,802 patients, that the use of prostate mpMRI decreased the number of patients undergoing a biopsy, decreased the detection of GS 6 PC, but increased the detection of GS 7 or higher.

We highlight its capability to spot high-grade cancerous lesions likely overlooked on regular biopsies, and in the process, guide targeted biopsies into suspicious lesions in the prostate (48) and consequently, the EAU PCa guidelines have adopted a first pre-biopsy MRI in the assessment of patients on AS for low-riskPCa (3).

##### 3.1.1.2 MRI and biopsy during AS

At any rate, MRI-targeted biopsies are as precise at identifying clinically significant diseases as systematic biopsies are and can do so with superior efficiency (49). When a positive MRI result is shown after a negative one in the initial biopsy, the tendency is to do a biopsy, preferably a target biopsy (2). Whereas, unfortunately, the sensitivity of TRUS-guided biopsy is not high, specifically when we deal with anterior tumours or large prostates, biopsies targeted to MRI lesions can identify violent PCa more accurately than standard TRUS-guided biopsies can (50). It is discussed whether mpMRI-targeted biopsies are sufficient to guarantee safety in patients who are under an AS protocol or if the addition of regular biopsies can be beneficial in accurate staging (48).

MRI-targeted biopsy is described as a procedure that uses the data obtained from mpMRI on the presence and position of a suspicious lesion at the moment of biopsy. The biopsy itself can be performed applying one of three methods, as stated by the START (Standards of Reporting for MRI-targeted biopsy Studies) collaborative group (49): (1) in-bore MRI TB, which is carried out in the MRI suite using real-time MRI guidance; (2) MRI-TRUS fusion TB where software is used to carry out an MRI and TRUS image fusion; and (3) cognitive registration TRUS TB, where the MRI is observed while carrying out the biopsy, and it is used cognitively to target the MRI-detected lesion applying TRUS guidance. Currently, there is no agreement as to which method of targeted biopsy ought to be chosen (51).

In men whose MRI results show a positive lesion, following targeted biopsy enhances patient risk in 40%–60% of cases (52). MRI-TB can be applied in two different diagnostic pathways: (1) the “combined pathway” where men with a positive mpMRI undergo combined regular and targeted biopsies, and men with a negative mpMRI only undergo regular biopsy and (2) the “MRI pathway” where men with a positive mpMRI undergo only MRI-TB, and men with a negative mpMRI do not undergo any biopsy. Magnetic resonance imaging-targeted biopsies considerably enhance the identification of ISUP grade ≥2 PCa and also identify fewer ISUP grade 1 cancers than systematic biopsies (3).

The PRECISION trial in 2018 was a prospective, randomised, multicentre trial whose aim was to compare a standard diagnostic pathway with an mpMRI-driven diagnostic pathway (53). In the mpMRI pathway, the patients underwent a fusion-TB of the suspicious lesion without an SB if they had an abnormal mpMRI (PI-RADS ≥3). The study determined that the mpMRI pathway provided a higher positive predictive value (PPV) and a negative predictive value (NPV) by reducing both the number of false positives and false negatives (54). The MRI-FIRST trial, a 2019 multicentre prospective trial, found that the detection of csPCa did not vary remarkably between SB and fusion-TB, while the detection rate of non-csPCa was remarkably higher by SB than fusion biopsy, suggesting a higher PPV for fusion-TB (3). These two trials indicate that the incorporation of SB to fusion-TB boosted the identification of csPCa and low-volume or low-grade tumours (55).

The ASIST study published by Klotz et al. (56) determined that regular biopsies ought to be performed despite the mpMRI findings, and the incorporation of MRI with targeted biopsies to regular biopsies did not remarkably increase the upgrading rate in comparison with regular biopsies alone. Nevertheless, the recently published 2-year post-biopsy follow-up report from the ASIST trial has revealed that the use of mpMRI before confirmatory biopsy ended in lesser progression to GG ≥2 PCa (57, 58). Finally, the EAU guidelines strongly advocate performing targeted and regular biopsies whenever the MRI result is positive (3).

##### 3.1.1.3 MRI during follow-up on AS

A consensus has not been reached either on if serial mpMRIs should be performed during AS or on their optimal regularity. In addition, it is still open to discussion if negative or stable mpMRI findings during follow-up could safely preclude follow-up biopsy. Prostate mpMRI as a monitoring tool for patients on AS is a developing field and categorising patients into either presenting “MRI progression” or “MRI stable/regression” could be helpful and could reduce the demand for repeating biopsies in some men (39).

The follow-up protocol is normally based on serial DRE (at least once a year), PSA (at least every 6 months), and repeated biopsies (3). Parameters that predict the risk of progression are mostly PSA kinetics, DRE, imaging such as mpMRI, and biomarkers such as prostate cancer antigen 3. The aim of reconsidering that patients undergo AS is to find the optimal moment to turn their attitude into active treatment (48).

Patients need to undergo surveillance biopsies during AS protocol to detect real disease progression and propose therapies with curative purposes. It is important to consider the use and timing of biopsy since the main risk of surveillance, together with the cancer itself, is the morbidity of prostate biopsy (59).

Although mpMRI has enhanced the selection of male patients for AS, it is relevant to point out that there is already enough evidence to maintain that imaging is not able to replace surveillance biopsies safely. MRIAS trial showed that a negative MRI could help exclude confirmatory biopsy but cannot be used to replace a 3-year surveillance biopsy because of the existence of imperceptible tumours (60). In 2013, a study retrospectively evaluated men selected for AS who had been given mpMRI and FB and the results showed that the number of lesions, lesion density, and highest mpMRI suspicion were prognostic of re-categorisation, so mpMRI could help the decision-making process for specialists regarding AS standard follow-up (61). In 2015, Diaz et al. determined that stable findings on mpMRI were prognostic of stable Gleason scores conveying fewer biopsies needed for men on AS (62).

On the other hand, Chestnut et al. suggested that both unchanged DRE and imaging stability do not preclude the necessity for systematic prostate biopsy. They highlighted that an AS protocol that requests repeating biopsy, only for alterations in clinical stage or MRI score, could liberate many patients from systematic biopsies but could miss many clinically significant PCa at the same time (63).

Giganti et al. found high disparities in measured tumour volume among patients with serial MRI. However, 88% of patients with no perceptible lesion on initial MRI had no targetable lesion at 3.6 years of follow-up (64).

Defining what constitutes radiologic progression on MRI is a key challenge. We should consider anatomic characteristics, namely, an alteration in size or stage; functional features, including DWI and dynamic contrast-enhanced MRI parameters, or a combination of findings concerning lesion visibility, as shown by PI-RADS scoring (2).

In 2016, the European School of Oncology arranged The Prostate Cancer Radiological Estimation of Change on Sequential Evaluation (PRECISE) panel to design guidelines for patients on AS for PCa. Their aim was to generate some guidance for divulging individual MRI studies in patients on AS and for investigators divulging the results of cohorts of patients undergoing MRI on AS (65). Applying a 1-to-5 scale for divulging the likelihood of radiologic progression (**Table 3**), the panel developed a reported proforma, which would be applied for each male patient and for each MRI, to gather the information in a standardised way (66).

**Table 3:** Evaluation of the likelihood of radiologic progression on magnetic resonance imaging in men on active monitoring (PRECISE score).

PRECISE score	Evaluation of the likelihood of radiologic progression
1	Resolution of previous features suspicious on MRI
2	Diminution in volume and/or visibility of features suspicious for prostate cancer
3	Stable MRI appearance: no new/focal diffuse lesions
4	Growth in size and/or visibility of features suspicious for prostate cancer
5	Definite radiologic stage progression (extraprostatic extension, seminal vesicle involvement, lymph node involvement, and metastasis)

Regarding progression criteria, it has been reported that mpMRI progression and pathologic upgrade are not associated. Nevertheless, higher baseline PI-RADS scores have been revealed to have an association with pathologic upgrading during monitoring in the AS population (67).

**Figure 2:**
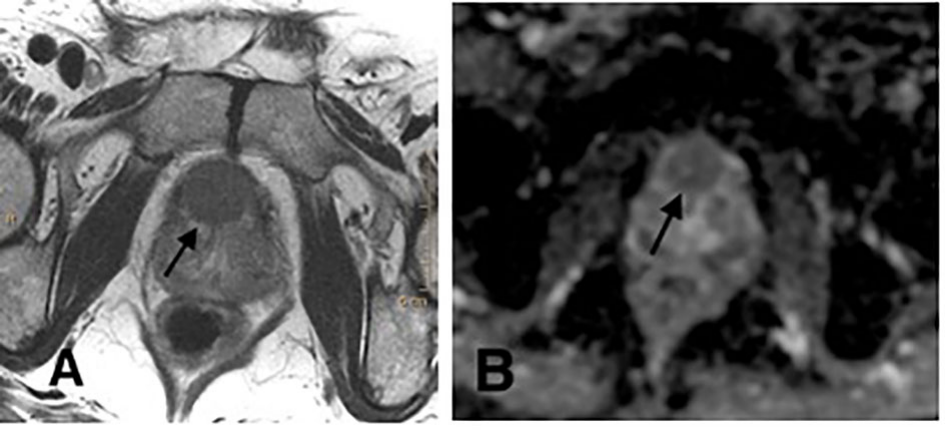
**(A)** Axial T2-weighted image with a hypointense lesion on the anterior transitional zone of the middle third bilaterally. **(B)** Axial diffusion-weighted image (ADC maps) with a marked diffusion restriction on the same zone. PI-RADS 5.

**Figure 2** is a patient with PCa ISUP grade 1 on AS protocol and who developed, on follow-up MRI, a PI-RADS 5 lesion, as we can see in the images. Subsequent perineal TB showed a PCa ISUP grade 2 with 5/5 positive cores on the target lesion. A radical treatment was proposed.

**Figure 3:**
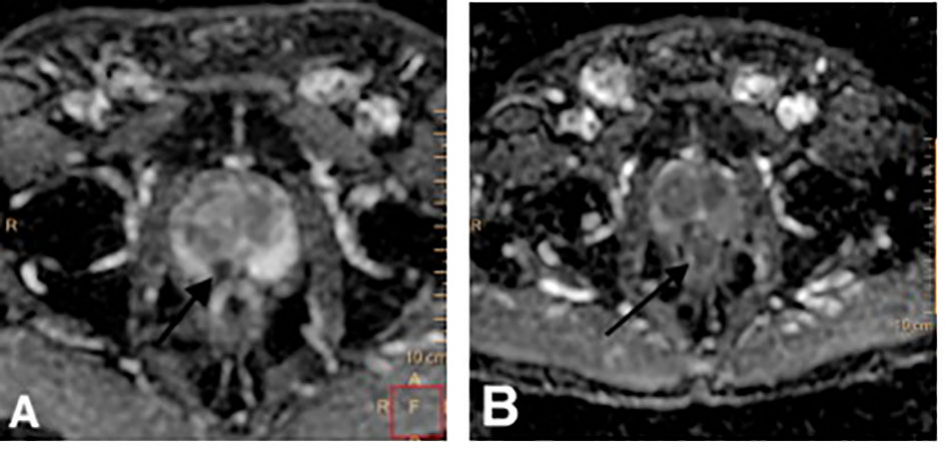
**(A, B)** Diffusion-weighted image (ADC maps). Marked diffusion restriction lesion on the posteromedial peripheric zone of the right middle third. The same lesion presents more diffusion restriction and has grown from **(A, B)**.

**Figure 3** is a patient with PCa ISUP grade 1 on AS protocol. On the control MRI, a PI-RADS 4 lesion is seen in the peripheric posteromedial region of the medial zone of the right prostatic lobe, which has progressed from the previous MRI. Subsequent TB showed PCa ISUP grade 2 with 4/5 positive cores in the target zone. The patient had radical prostatectomy.

AS programs for male patients typically have tighter follow-up in the early period to detect potential miscategorisation at diagnosis. There is ongoing debate over the use of PSA kinetics in men on AS, with some communications indicating that PSA kinetics is not prognostic of an upgraded rate after biopsy. However, Giganti et al. (68) demonstrated in 2020 that the alteration in PSA density was associated with radiologic progression. Their study also concludes that identifying men on AS with progression (PRECISE 3–4) promotes re-biopsy/treatment and avoids repeating biopsy in patients with PRECISE 1–2, shortening over-monitoring for the individual and the healthcare system.

The experience with misattribution and undergrading described in the surveillance literature largely reflects the pre-MRI era. Increasingly, patients on surveillance undergo an MRI and targeted biopsy. The rate of undergrading is much less with targeted biopsy than with systematic biopsy (69, 70). This should have many positive benefits, including earlier identification of aggressive high-grade cancer; for patients with a negative MRI or targeted biopsy, reassurance that their risk of harbouring high-grade cancer is low; and a more accurate signal for intervention (1).

Two years ago, our centre began its participation in the PRECISE (Prostate Cancer Radiological Estimation of Change in Sequential Evaluation) study, which involves 22 centres in total. It uses two inclusion criteria: patients with at least two mpMRIs (at diagnosis and during follow-up) and patients with at least two prostate biopsies. Local radiologists subsequently reported mpMRI according to PRECISE, and any increase in the Gleason score denotes histologic progression. So far, our centre has participated with 37 patients with an average follow-up time of 32 months. Thirty-four (92%) of our patients had an ISUP grade 1 PCa on baseline biopsy and 48.6% (18) resulted in a global PRECISE 4 after two mpMRI, the rest being PRECISE 3. Eight (21.6%) of our patients discontinued the protocol because of PSA and mpMRI progression. Due to our low sample, overall survival results have not been analysed (71). These multicentre study results need to be evaluated, and their publication will help us continue learning about the function of mpMRI in AS, the standard treatment for low-risk prostate cancer.

## 4 Conclusions

Being aware of the risk of miscategorisation of PCa risk groups during the first SB, MRI has certainly become nearly the standard, especially because of its high NPV for lesion upgrading and its ability to locate lesions, particularly in the anterior area of the prostate. This has decreased the risk of initial misclassification in different AS series, and this result contributes to the increasing use of mpMRI during follow-up. Moreover, having negative MRI results could restrict the number of future biopsies in male patients with stable-disease. On the other hand, new lesions or changes in previous ones might indicate disease progression and as such, TB becomes mandatory. In the future, the addition of novel and promising biomarkers to imaging will allow us to join the era of precision medicine in uro-oncology, especially in cases of PCa.

## Author contributions

ID: Conceptualization, Data curation, Investigation, Methodology, Resources, Software, Supervision, Validation, Writing – original draft, Writing – review & editing. JG: Conceptualization, Data curation, Investigation, Methodology, Resources, Software, Supervision, Validation, Writing – original draft, Writing – review & editing. BG: Conceptualization, Data curation, Supervision, Visualization, Writing – review & editing. MM: Conceptualization, Data curation, Writing – review & editing. DE: Investigation, Software, Supervision, Writing – review & editing. BS: Investigation, Methodology, Software, Supervision, Writing – review & editing. JB: Data curation, Resources, Supervision, Visualization, Writing – review & editing. JM-S: Conceptualization, Supervision, Writing – review & editing.
